# Supplementary material for: Genetic comparisons of interleukin-17 reveal a framework for complex signaling evolution
Source: bioRxiv. 2026 Apr 14:2026.04.13.718218. Preprint. [Version 1] doi: 10.64898/2026.04.13.718218 (PMC13105070; doi:10.64898/2026.04.13.718218)
Supplement: Supplement 3 [file media-3.zip › Supplemental files for paper/ERC/IL17xIL17_comparisons/IL17REL_IL17REL.pdf]

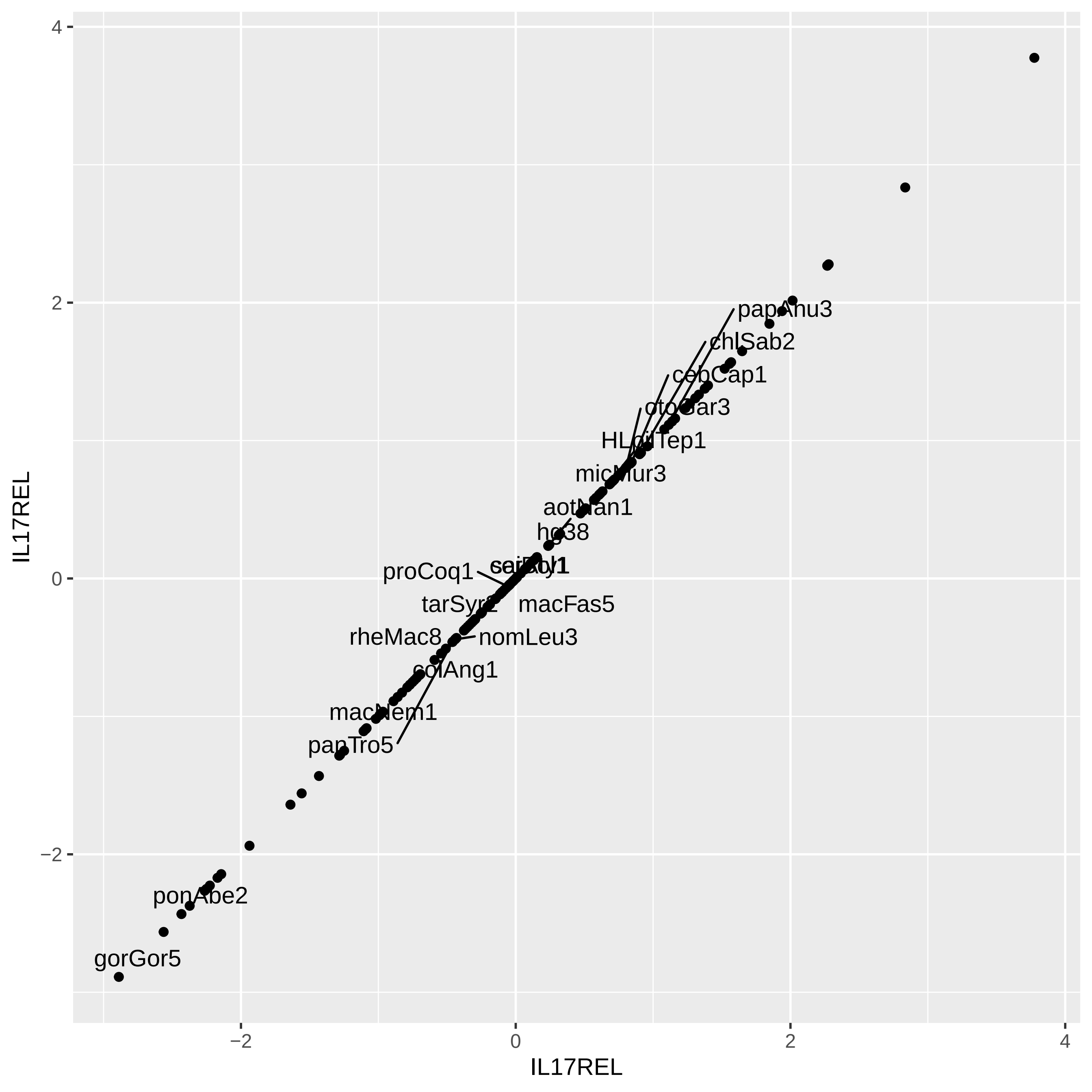

papAnu3
chlSab2
cebCap1
otoGar3
HLpilTep1
micMur3
aotNan1
hg38
proCoq1
tarSyr2
macFas5
rheMac8
nomLeu3
colAng1
macNem1
panTro5
ponAbe2
gorGor5
IL17REL
IL17REL
4
2
0
−2
−2
0
2
4
